# Editorial: Non-neuronal cells in neurodegenerative diseases and psychiatric disorders

**DOI:** 10.3389/fncel.2025.1623523

**Published:** 2025-06-09

**Authors:** Enrique Estudillo, Gilberto Pérez-Sánchez, Lenin Pavón, Adriana Jiménez

**Affiliations:** ^1^Laboratorio de Reprogramación Celular, Instituto Nacional de Neurología y Neurocirugía Manuel Velasco Suárez, Ciudad de México, México; ^2^Laboratorio de Psicoinmunología, Instituto Nacional de Psiquiatría Ramón de la Fuente Muñiz, Ciudad de México, México; ^3^División de Investigación, Hospital Juárez de México, Ciudad de México, México

**Keywords:** astrocyte, oligodendrocyte, microglia, neurobiology of disease, physiology, pathophysiology, psychiatry, stem cell

Recent studies on non-neuronal cells have revealed their critical role in maintaining brain homeostasis through complex interactions with neurons. These findings suggest that non-neuronal cells are essential for understanding neurodegenerative diseases and psychiatric disorders, warranting further investigation. This Research Topic aims to explore how non-neuronal cells contribute to neurodegeneration and psychiatric illnesses.

In their study, Quan et al. provided new insights into the role of the NLR family pyrin domain containing 3 protein (NLRP3) in Alzheimer's disease by examining the effects of its deletion on pericytes in a mouse model of this neurodegenerative condition. These results indicate that NLRP3 plays a significant role in disease pathophysiology by promoting a protective effect on pericytes when it is ablated, suggesting that NLRP3 could be a potential therapeutic target for Alzheimer's disease.

Manickam et al. investigated the relationship between synaptic repair and the survival of spiral ganglion neurons following noise-induced cochlear synaptopathy (NICS) and reported that macrophages may play a crucial role. Their study revealed that the soluble chemokine fractalkine can restore ribbon synapses and hearing post-NICS by regulating cochlear macrophage numbers and reducing inflammation. Notably, the beneficial effects of fractalkine on cochlear synaptopathy were dependent on the presence of macrophages; without these effects, fractalkine failed to restore lost synapses and hearing. These findings underscore the importance of macrophages in recovery from hearing damage.

Depression, a mood disorder, is associated with various environmental and genetic risk factors. Treatment-resistant depression often correlates with elevated inflammatory markers, leading to the hypothesis that neuroinflammation may trigger comorbid depression. Neuroinflammation is defined as the immune response in the brain and spinal cord that involves the activation of glia and the release of a variety of inflammatory mediators. Jia et al. explored whether the axis between the gut microbiota and Th17 cells influences the development of inflammatory depression. Their research indicated that gut dysbiosis, along with increased levels of peripheral T helper 17 cells (Th17) and interleukin-17A (IL-17A), could contribute to depressive symptoms. Additionally, the microbiota appears to stimulate peripheral immune cells, promoting their migration to the central nervous system (CNS) and resulting in depressive-like behaviors in animal models. However, some findings remain controversial, suggesting the need for further studies to clarify the role of the gut microbiota-Th17 cell axis in CNS inflammation and its relationship with depression.

Using bioinformatics tools, Kondo et al. analyzed data from white matter cells of patients with schizophrenia and discovered an enrichment of schizophrenia-associated genetic risk variants in oligodendrocyte precursor cells, which are linked to synaptic processes. These significant findings reinforce the vital role of oligodendrocyte lineage cells in psychiatric disorders and highlight the complex contributions of oligodendrocyte precursors to the physiology of the CNS.

Microglia, the specialized immune cells of the CNS, have essential functions in both adult and developing brains, yet their properties can change under pathological conditions. While the role of microglia in aging and neurodegenerative diseases is well documented, maternal immune activation from environmental stressors and infections may increase the risk of microglial reactivity, contributing to neurodevelopmental disorders. In this context, Shimamura et al. presented recent advances in understanding how disruptions of microglia during maternal immune activation can impair brain development and lead to neurodevelopmental disorders.

Many human psychiatric disorders remain poorly understood due to a lack of models that effectively study the cellular properties and neurobiological mechanisms involved. Villafranco et al. presented a narrative review discussing how glial and neuronal cells derived from induced pluripotent stem cells (iPSCs) of patients with depression can help to elucidate the molecular mechanisms behind this disorder. Their work underscores promising findings regarding the phenotypic properties of neurons and non-neuronal cells from depression patients, encouraging further research using these platforms to increase our understanding of the disorder.

Vijayaraghavan et al. emphasized the importance of iPSCs in studying the phenotypic properties of glial cells in hereditary spastic paraplegias. This review provides valuable insights into the importance of lipid metabolism in non-neuronal cells, particularly astrocytes, for maintaining brain integrity. The narrative also highlights the role of microglia in this neurodegenerative condition and how iPSCs have contributed to the progress of our knowledge about the relationship between microglia and hereditary spastic paraplegia.

Understanding psychiatric and neurodegenerative diseases is complex and requires innovative approaches. There is a need to explore non-conventional technologies as alternative therapeutic strategies, especially given the limited effectiveness of current treatments. Villanueva, provides a valuable narrative that highlights recent advances in the use of stem cells as therapeutic agents for psychiatric disorders. Although the application of stem cells in psychiatric treatments may seem far-fetched at the moment, there is significant potential for improvement in this area.

This Research Topic aims to inspire the scientific community to further investigate and enhance therapeutic strategies that utilize stem cells or their derivatives for psychiatric conditions. Furthermore, this Research Topic presents updated and novel findings that encourage new approaches to understand and study neurodegenerative diseases and psychiatric disorders. Effective and comprehensive management of these health issues can be achieved only by recognizing non-neuronal cells as essential components of their pathophysiology.

